# Cost-Effectiveness of Total Colonoscopy in Screening of Colorectal Cancer in Japan

**DOI:** 10.1155/2012/728454

**Published:** 2012-01-12

**Authors:** Masau Sekiguchi, Takahisa Matsuda, Naoto Tamai, Taku Sakamoto, Takeshi Nakajima, Yosuke Otake, Yasuo Kakugawa, Yoshitaka Murakami, Yutaka Saito

**Affiliations:** ^1^Endoscopy Division, National Cancer Center Hospital, 5-1-1 Tsukiji, Chuo-ku, Tokyo 104-0045, Japan; ^2^Cancer Screening Division, Research Center for Cancer Prevention and Screening, National Cancer Center, Tokyo 104-0045, Japan; ^3^Department of Medical Statistics, Shiga University of Medical Science, Shiga 520-2192, Japan

## Abstract

*Introduction*. In Japan, the cost-effectiveness of total colonoscopy (TCS) for primary screening of colorectal cancer (CRC) is unclear. We compared the cost of identifying a patient with CRC using two primary screening strategies: TCS (strategy 1) and the immunochemical fecal test (FIT) (strategy 2). *Materials and Methods*. We retrospectively analyzed the TCS screening database at our institution from February 2004 to August 2010 (strategy 1, *n* = 15,348) and the Japanese nationwide survey of CRC screening in 2008 (strategy 2, *n* = 5,267,443). *Results*. 112 and 6,838 CRC cases were detected in strategies 1 and 2, costing 2,124,000 JPY and 1,629,000 JPY, respectively. The rate of earlier-stage CRC was higher in strategy 1. *Conclusions*. The cost was higher using TCS as a primary screening procedure. However, the difference was not excessive, and considering the increased rate of detecting earlier CRC, the use of TCS as a primary screening tool may be cost-effective.

## 1. Introduction

In Japan, the incidence and mortality rate of colorectal cancer (CRC) has increased significantly, with an incidence of approximately 100,000 cases and over 40,000 deaths per year [1]. CRC is now the second most commonly diagnosed cancer and the third leading cause of cancer-related mortality in Japan. In order to decrease the incidence and mortality of CRC, a screening system has been established. There are two types of CRC screening in Japan; one is population-based screening recommended for the entire population aging 40 and over, and the other is opportunistic screening. In population-based screening, the immunochemical fecal test (FIT) is used as a primary screening tool and total colonoscopy (TCS) is only performed for those with a positive FIT. TCS is not used as a primary screening procedure in population-based screening. On the other hand, in opportunistic screening, TCS is widely used as a primary screening procedure.

In this situation, the relative cost-effectiveness of different CRC screening strategies needs to be clarified. Such analyses have been performed in the United States and other countries [2–8], but in Japan, there have been limited analyses of the cost-effectiveness of CRC screening [9, 10], with the studies available demonstrating the population-based screening strategy to be cost-effective. In contrast, the cost-effectiveness of TCS as a primary screening strategy in opportunistic screening is still unclear.

In this study, our primary objective was to compare the cost of identifying a patient with CRC in Japan using two strategies: TCS as a first screen (strategy 1) versus FIT as a first screen (strategy 2).

## 2. Materials and Methods

We retrospectively analyzed the cost of identifying a patient with CRC using strategies 1 and 2 as follows.

### 2.1. Strategy 1: TCS as a Primary Screening

We retrospectively reviewed the database of the Cancer Screening Division, Research Center for Cancer Prevention and Screening, National Cancer Center, which followed all subjects given a TCS as a primary screening from February 2004 to August 2010. A total of 15,348 cases had a colonoscopy performed as a primary screening. This data was used to calculate the cost associated with identifying a patient with CRC using the cost of TCS as 15,500 JPY, based on Japanese national reimbursement tables.

### 2.2. Strategy 2: FIT as a Primary Screening

We retrospectively analyzed the Japanese nationwide survey of CRC screening in 2008 [11]. A total of 5,267,443 cases were included. This data was used to calculate the cost associated with identifying a patient with CRC using the cost of FIT as 1,600 JPY and TCS as 15,500 JPY, respectively.

## 3. Results

Clinical characteristics of examinees in strategies 1 and 2 are listed in Table 1. Both groups predominantly comprised examinees in their 50s and 60s, and there was a higher male-to-female ratio in strategy 2 than in strategy 1. However, there was no statistical significance between two groups.

The number of CRC cases identified and the cost to find one case of CRC in both groups are listed in Table 2. In strategy 1, there were 112 cases of CRC among 15,348 TCS examinees (0.73%), with a calculated cost of finding one CRC case of 2,124,000 JPY. In group 2, 5,267,443 underwent FIT, with 319,846 cases testing positive, (6.1%). All examinees with a positive FIT were recommended for a further TCS. However, only 174,914 examinees (54.7%) underwent TCS, and 6,838 cases of CRC were found. The calculated cost to find one patient with CRC was 1,629,000 JPY in this group. If all of the 319,846 cases with a positive FIT had undergone TCS, the number of CRC cases would have increased, reducing the cost of identifying CRC. Assuming that the rate of CRC cases among the TCS examinees was the same as that in the strategy 2 group (3.9%; 6,838/174,914), it was calculated that there would be 12,504 CRC patients, each costing 1,090,000 JPY to be identified.

The staging of CRC at diagnosis (Japanese Classification of Colorectal Carcinoma) and initial treatment for CRC are summarized in Table 2. The rate of stage 0 and endoscopic resection were higher in strategy 1 than in strategy 2.

## 4. Discussion

Several previous studies have shown that CRC screening including FIT and TCS is cost-effective. However, in Japan, only a few cost-effective analyses have been reported, with the cost-effectiveness of TCS as primary screening still unclear.

**Figure 1:**
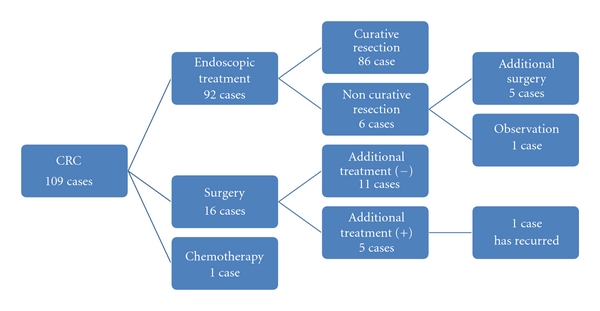
The clinical course of CRC cases detected in strategy 1.

In this analysis, we compared the cost of identifying a patient with CRC using two screening strategies, using TCS as a primary screening, or using FIT as a primary screening with TCS then performed in cases with a positive FIT test. The results demonstrated that it cost more to identify CRC when TCS was used as a primary screening strategy compared to the FIT screening strategy (2,124,000 JPY versus 1,629,000 JPY). It is assumed that this difference would have become even larger if all FIT-positive subjects had then chosen to have a TCS (2,124,000 JPY versus 1,090,000 JPY). However, the higher cost associated with the TCS only strategy does not necessarily deny the cost-effectiveness of this approach for primary screening. This is because TCS, used as a primary screening strategy, was able to identify CRC at an earlier stage as demonstrated in Table 2, possibly resulting in a decreased cost of CRC treatment and followup. The clinical course of the cases of CRC detected in strategy 1 at our institution is shown in Figure 1. Among the 112 CRC cases identified, 109 cases followed a clear clinical course, with approximately 80% cured with a single endoscopic treatment. Only one case has had recurrent disease following treatment. Such a clinical course indicates that earlier detection of CRC can lead to cure with less invasive treatment, resulting in a shorter period of followup and decreased cost of CRC care. From this perspective, it is possible to postulate that the difference in the cost of identifying CRC in the two strategies is not as great and that TCS may be a cost-effective primary screening strategy. Additionally, we probably underestimated the cost-effectiveness of TCS because we did not include the possibility to reduce CRC incidence with TCS in this study. Previous studies have demonstrated the effect of colonic polypectomy in reducing CRC incidence [12, 13]. Not only when using TCS as a primary screening strategy but also when using FIT as a primary screening, reduction in CRC incidence is expected [14]. However, taking into account the higher detection rate for colorectal polyps with TCS and the low rate of undergoing TCS among examinees with a positive FIT, reduction in CRC incidence is expected more when using TCS as a primary screening. If we consider this effect of TCS, TCS may be a more acceptable choice as a primary screening. Furthermore, considering that using TCS as a primary screening can lead to better quality of life (QOL) after CRC diagnosis due to the earlier detection of disease, it is worth performing TCS as a primary screening of CRC.

## 5. Conclusions

The cost associated with identifying one case of CRC is higher when using TCS as a primary screening strategy compared to using the FIT as a primary screening. However, taking into account the earlier detection of CRC using TCS, it is possible to postulate that the final cost difference may be reduced and that TCS may provide a cost-effective primary screening strategy. Additionally, considering the effect of TCS on CRC incidence and a better QOL after earlier detection of CRC with TCS, TCS is worth using as a primary screening of CRC.

## Figures and Tables

**Table 1:** Clinical characteristics of examinees in strategies 1 and 2.

	Strategy 1 (*n* = 15, 348)	Strategy 2 (*n* = 5, 267,443)
Screening strategy	TCS as a primary screening	FIT as a primary screening
Sex		
Male	5,892 (38.4%)	2,174,604 (41.3%)
Female	9,456 (61.6%)	2,006,926 (38.1%)
Unknown	0	1,085,913 (20.6%)
Age group (yr)		
<40	15 (0.1%)	370,750 (7.0%)
40–49	1,918 (12.5%)	870,134 (16.5%)
50–59	4,864 (31.7%)	1,050,813 (19.9%)
60–69	6,521 (42.5%)	1,044,313 (19.8%)
≧70	2,030 (13.2%)	845,520 (16.1%)
Unknown	0	1,085,913 (20.6%)
Mean (range)	60.1 (40–89)	Unknown

**Table 2:** Number of CRC cases, the cost to find one CRC case, staging of CRC at diagnosis, and initial treatment for CRC in both strategies.

	Strategy 1 (*n* = 15, 348)	Strategy 2 (*n* = 5, 267,443)
Number of cases of CRC	112 (0.73%)	6,838 (0.13%)
Cost to find a case of CRC	2,124,000 JPY	1,629,000 JPY
Staging of CRC at diagnosis		
0	81 (72.3%)	1,713 (25.1%)
I	16 (14.3%)	1,043 (15.3%)
II	7 (6.3%)	552 (8.1%)
III a	3 (2.7%)	418 (6.1%)
III b	1 (0.9%)	187 (2.7%)
IV	1 (0.9%)	116 (1.7%)
Unknown	3 (2.7%)	2,809 (41.1%)

Initial treatment for CRC		
Endoscopic treatment	93 (83.0%)	2,267 (33.2%)
Surgery	16 (14.3%)	2,466 (36.1%)
No treatment	0	19 (0.3%)
Others	0	67 (1.0%)
Unknown	3 (2.7%)	2,019 (29.5%)

## References

[1] Matsuda T, Marugame T, Kamo K-I, Katanoda K, Ajiki W, Sobue T (2011). Cancer incidence and incidence rates in Japan in 2005: based on data from 12 population-based cancer registries in the monitoring of cancer incidence in Japan (MCIJ) project. *Japanese Journal of Clinical Oncology*.

[2] Frazier AL, Colditz GA, Fuchs CS, Kuntz KM (2000). Cost-effectiveness of screening for colorectal cancer in the general population. *JAMA*.

[3] Sonnenberg A, Delcò F, Inadomi JM (2000). Cost-effectiveness of colonoscopy in screening for colorectal cancer. *Annals of Internal Medicine*.

[4] Pignone M, Saha S, Hoerger T, Mandelblatt J (2002). Cost-effectiveness analyses of colorectal cancer screening: a systematic review for the U.S. Preventive Services Task Force. *Annals of Internal Medicine*.

[5] Song K, Fendrick AM, Ladabaum U (2004). Fecal DNA testing compared with conventional colorectal cancer screening methods: a decision analysis. *Gastroenterology*.

[6] Vijan S, Hwang I, Inadomi J (2007). The cost-effectiveness of CT colonography in screening for colorectal neoplasia. *American Journal of Gastroenterology*.

[7] Tsoi KKF, Ng SSM, Leung MCM, sung JJY (2008). Cost-effectiveness analysis on screening for colorectal neoplasm and management of colorectal cancer in Asia. *Alimentary Pharmacology and Therapeutics*.

[8] Lansdorp-Vogelaar I, Knudsen AB, Brenner H (2011). Cost-effectiveness of colorectal cancer screening. *Epidemiologic Reviews*.

[9] Tsuji I, Fukao A, Shoji T, Kuwajima I, Sugawara N, Hisamichi S (1991). Cost-effectiveness analysis of screening for colorectal cancer in Japan. *Tohoku Journal of Experimental Medicine*.

[10] Shimbo T, Glick HA, Eisenberg JM (1994). Cost-effectiveness analysis of strategies for colorectal cancer screening in Japan. *International Journal of Technology Assessment in Health Care*.

[11] (2011). Nationwide Survey Committee of Mass Screening for Digestive Organs of the Japanese Society of Gastroenterological Cancer Screening: annual report 2008 of the nationwide survey on mass screening for digestive organs. *Journal of Gastroenterol Cancer Screening*.

[12] Winawer SJ, Zauber AG, May Nah Ho (1993). Prevention of colorectal cancer by colonoscopic polypectomy. *The New England Journal of Medicine*.

[13] Citarda F, Tomaselli G, Capocaccia R, Barcherini S, Crespi M (2001). Efficacy in standard clinical practice of colonoscopic polypectomy in reducing colorectal cancer incidence. *Gut*.

[14] Mandel JS, Church TR, Bond JH (2000). The effect of fecal occult-blood screening on the incidence of colorectal cancer. *The New England Journal of Medicine*.

